# Psychological Contracts and Emotional Labor in the Age of AI: A Moderated Mediation Model

**DOI:** 10.3390/bs16060918

**Published:** 2026-06-03

**Authors:** Kübra Karakış, Oya Erdil

**Affiliations:** Department of Business Administration, Faculty of Business Administration, Gebze Technical University, 41400 Kocaeli, Türkiye; erdil@gtu.edu.tr

**Keywords:** psychological contract, AI anxiety, general attitudes toward AI, generative AI acceptance, emotional labor

## Abstract

This study examines how employees’ perceptions of transactional and relational psychological contracts influence emotional labor strategies in contemporary work contexts where AI technologies are increasingly present through AI anxiety, general attitudes toward AI, and generative AI acceptance. Based on Conservation of Resources Theory, Cognitive Appraisal Theory, the Theory of Planned Behavior and technology acceptance frameworks (UTAUT), a conceptual model was tested using survey data from 869 employees across various sectors in Türkiye. Mediation and moderated mediation analyses were conducted using the PROCESS macro for SPSS 30. The results showed that transactional psychological contracts were positively correlated with surface acting, while relational psychological contracts were associated with deep acting. Serial mediation analyses indicated that relational psychological contracts were indirectly associated with higher levels of deep acting, primarily through more positive evaluations of AI, with the full sequential pathway through anxiety reduction not operating as hypothesized. Generative AI acceptance mediated the relationship between negative attitudes toward AI and surface acting. Moreover, generative AI acceptance mediated the relationship between positive attitudes toward AI and deep acting, indicating a pathway through which favorable technology evaluations translate into authentic emotional regulation. Finally, moderated mediation analyses suggest that emotional intelligence strengthens the impact of generative AI acceptance on employees’ emotional labor strategies.

## 1. Introduction

The rapid integration of artificial intelligence (AI) into organizational systems is transforming how work is structured, supervised, and experienced. AI-driven technologies now perform tasks previously considered uniquely human, creating new uncertainties regarding control, autonomy, and the continuity of professional roles. Although AI can enhance productivity, its increasing autonomy may also generate emotional strain and concerns about employees’ positions in technology-intensive workplaces (Akkaya et al., 2021; Wang & Wang, 2022). Because many employees are still unfamiliar with AI systems, forming stable expectations about their use and outcomes remains difficult (Kaya et al., 2024).

When AI becomes part of daily work, employee responses include both emotional and cognitive reactions that shape work behavior. Research shows AI anxiety is linked to burnout, resistance, and lower commitment (X. Chen et al., 2025). Despite this growing interest, AI anxiety is rarely studied as a key mechanism in broader emotional-cognitive models of employee behavior (Liu et al., 2025). More fundamentally, the emotional and relational foundations behind employee responses to AI require greater theoretical attention.

AI adoption may also reshape how employees interpret their relationship with the organization. Psychological contract theory conceptualizes employment relationships based on mutually perceived obligations between employees and organizations (Rousseau, 1995). However, the increasing use of AI in organizational decision processes may alter these perceptions. Employees may interpret AI adoption as either a supportive technological change or a potential threat to job security and future career opportunities, which can influence how they evaluate organizational obligations (Cheng et al., 2025).

Artificial intelligence may create both resource gains and losses for employees. Prior research suggests that AI can reduce routine workloads while simultaneously increasing performance pressure and concerns about skill obsolescence (Lin & He, 2025). These dynamics shape how employees interpret AI-related experiences in terms of potential opportunities and threats, giving rise to both cognitive evaluations and emotional responses. AI-related anxiety represents a distinct psychological reaction to technological uncertainty, often reflecting concerns about job replacement and the future relevance of human skills (Shekhar & Saurombe, 2026).

Employees’ responses to artificial intelligence can be understood in terms of how evaluative processes translate into behavioral tendencies (Ajzen, 1991). Individuals form general evaluations of AI based on their assessments of its potential benefits and risks, while feelings of anxiety reflect an affective reaction to perceived uncertainty and threat (Wang & Wang, 2022). Together, these cognitive and emotional responses emerge from employees’ interpretations of technology-related experiences and shape their behavioral orientations in work contexts.

Although prior research has shown that psychological contracts influence emotional labor, limited attention has been given to how these relationships operate in settings where employees engage with intelligent technologies. In particular, the roles of evaluative judgments, AI anxiety responses, and generative AI acceptance in shaping emotional labor strategies remain underexplored. To address this gap, the present study examines how transactional and relational psychological contracts influence employees’ emotional labor strategies in the context of individual interactions with AI. The model investigates the mediating roles of general attitudes toward AI, AI anxiety, and generative AI acceptance, as well as the moderated mediating role of perceived emotional intelligence, in linking contract orientations to emotional labor outcomes.

### 1.1. Psychological Contract

The term psychological contract was first used in industrial psychology by Argyris (1960) as the psychological labor contract to denote the invisible, implicit relationships between employers and employees in factory settings. The psychological contract concept is defined as “an individual’s beliefs regarding the terms of a mutual exchange agreement between a focal person and another party” (Rousseau, 1989, p. 123). Psychological contracts are commonly categorized into two types: transactional contracts, which are short-term and primarily based on economic exchange, and relational contracts, which are long-term and emphasize socioemotional elements such as loyalty and mutual commitment (Rousseau, 1989).

Recent technological developments, particularly the rapid adoption of AI, are transforming how these psychological contracts operate in organizations (Bankins & Formosa, 2021). The rise in algorithmic management introduces AI as an influential actor in organizational decision-making processes, including recruitment, performance evaluation, and task allocation (Bankins & Formosa, 2020; Tomprou & Lee, 2022). As AI systems increasingly participate in these functions, they alter the relational dynamics that traditionally define the exchange between employees and organizations.

‘Employees’ resource-related perceptions in work contexts where AI technologies are increasingly visible are closely linked to how psychological contract orientations shape their cognitive and emotional responses to technological change (Khairy et al., 2025; Kim et al., 2024; Moin et al., 2024).

### 1.2. General Attitudes Toward AI

Attitude toward AI refers to an individual’s overall positive or negative evaluation of AI-based systems and applications. Prior research shows that these attitudes are significantly associated with individuals’ willingness to adopt and use AI technologies (Buck et al., 2022; Dong et al., 2024; Aktulun et al., 2024). Attitudes toward AI play an important role in shaping individuals’ intentions and behaviors regarding emerging technologies (Katsantonis & Katsantonis, 2024). The Theory of Planned Behavior (TPB) suggests that these attitudes influence behavioral orientations through evaluative and motivational processes (Ajzen, 1991).

General attitudes toward AI are commonly conceptualized as consisting of two dimensions, positive and negative attitudes, measured through the General Artificial Intelligence Attitude Scale (GAAIS) developed by Schepman and Rodway (2020). Unlike attitudes toward specific AI applications, general AI attitudes capture broader evaluations of AI as a transformative technology with social, ethical, and functional implications. Positive attitudes reflect beliefs that AI can create economic opportunities and improve work processes, while negative attitudes are associated with concerns about ethical risks, loss of control, and potential misuse of AI systems (Kavak, 2024). In this context, employees’ attitudes toward AI may be relevant to how they manage their emotional responses in work settings where AI is present (Konuk et al., 2023).

### 1.3. AI Anxiety

The origins of artificial intelligence anxiety can be traced back to the early emergence of modern computing, when scholars began to question whether thinking machines might eventually replace human cognition (D. G. Johnson & Verdicchio, 2017). AI anxiety refers to the fear and unease associated with the perceived loss of control over artificial intelligence systems (Wang & Wang, 2022; Akkaya et al., 2021). This anxiety develops when individuals associate AI-related cues such as automation, algorithms, or autonomous decision-making with potential threats to their professional competence, job security, and sense of control, thereby triggering generalized apprehension across both personal and professional contexts (J. Li & Huang, 2020; Wang & Wang, 2022).

The construct is commonly conceptualized as multidimensional, including learning anxiety, job displacement anxiety, sociotechnical blindness, and AI configuration anxiety (Wang & Wang, 2022). Learning anxiety refers to concerns about acquiring the skills needed to understand AI systems or complex algorithms to remain professionally competitive (Terzi, 2020; Hopcan et al., 2024). Job displacement anxiety reflects fears that AI systems and humanoid robots may replace human labor, leading to job insecurity. Sociotechnical blindness describes misconceptions about the autonomy of AI systems and the belief that AI may operate independently of human oversight (D. G. Johnson & Verdicchio, 2017). Finally, AI configuration anxiety arises from perceptions of opacity and unpredictability in AI systems, which can evoke feelings of intimidation and loss of control (Hopcan et al., 2024).

AI anxiety should be distinguished from traditional forms of computer anxiety or general technology-related stress. Conventional technology anxiety typically stems from individuals’ concerns about their ability to operate a particular system or device. In contrast, AI anxiety is linked to the perceived autonomy and opacity of AI systems, often referred to as the “black box” problem, as well as concerns about the broader social and ethical implications of intelligent technologies (D. G. Johnson & Verdicchio, 2017; Wang & Wang, 2022). AI systems differ from conventional software in that they may replace not only physical labor but also aspects of human judgment and decision-making, thereby extending their impact beyond that of standard organizational tools (J. Li & Huang, 2020). Concerns about the loss of human control over intelligent systems and the possibility of ethical violations in AI-driven decisions further reinforce AI anxiety as a distinct form of technological threat perception (Wang & Wang, 2022; J. Li & Huang, 2020).

Empirical findings reveal that AI anxiety exerts complex influences on human attitudes and behaviors. For instance, Öztırak (2023) found that moderate levels of AI anxiety may enhance innovation-oriented behavior by motivating employees to adapt to technological change. However, excessive anxiety tends to undermine optimism and positive job perceptions (S. Chen & Zhao, 2025). Research on pre-service teachers demonstrated that female participants reported higher anxiety levels than males in learning, job displacement, and AI configuration dimensions, though no significant gender difference emerged in sociotechnical blindness (Terzi, 2020).

Empirical research on AI anxiety remains limited in explaining how this construct relates to employees’ psychological contracts and emotional regulation strategies. This limitation restricts a comprehensive understanding of how affective responses to AI shape workplace adaptation and human–AI interaction processes. In the present study, AI anxiety is conceptualized as a technology-related evaluative apprehension that contains affective elements but functions within the broader cognitive appraisal process, consistent with its role as a sequential mediator in the proposed model.

### 1.4. Emotional Labor

Emotional labor is defined as “the management of feeling to create a publicly observable facial and bodily display” (Hochschild, 1983, p. 7). In the literature, emotional labor is typically conceptualized through two primary strategies: surface acting and deep acting. Surface acting refers to modifying observable emotional expressions without changing one’s internal emotional state to meet organizational expectations. In contrast, deep acting involves intentionally regulating one’s internal emotional state to genuinely experience the emotions required by the organization (Grandey, 2000).

Emotional labor refers to the management of feelings to meet organizational display expectations and has been conceptualized as a behavioral response to work-related affective events (Weiss & Cropanzano, 1996), which links workplace events to employees’ emotional responses and regulation processes. Organizational display rules shape how employees manage their emotions, and these rules are increasingly influenced by technological systems such as algorithmic feedback mechanisms and digital monitoring tools in contemporary work environments where AI tools are becoming more prevalent.

The rapid development of AI systems in the digital era is transforming both the emotional expectations placed on employees and the ways in which emotional labor is performed in organizations. AI-driven automation and digital monitoring can intensify standardization and control over work processes, potentially increasing emotional dissonance in occupations that require high levels of emotional labor, such as call center work (Konuk et al., 2023). For instance, large organizations such as Walmart employ emotion recognition technologies to monitor customer reactions and adjust service processes accordingly (Haenlein et al., 2019). At the same time, the introduction of AI-based monitoring systems may affect employees’ perception of trust within the organization. Continuous digital observation can create the impression that employers do not trust employees, which may weaken the employee–organization relationship. Moreover, concerns about being digitally recorded may discourage open communication and reduce social support among coworkers, thereby affecting workplace relationships (Roemmich et al., 2023). AI therefore shapes not only the tasks employees perform but also how these tasks are carried out. This effect becomes particularly visible in occupations that require frequent emotional interaction with customers, such as customer support roles (Oder & Béland, 2025).

Emotional labor in work contexts characterized by growing AI presence can be understood as a process in which employees continuously balance emotional authenticity with adaptive emotional regulation, shaped by both individual capabilities and technological work conditions.

### 1.5. Generative AI Acceptance

Generative AI acceptance refers to an individual’s or organization’s intention to use or integrate AI systems capable of generating new and creative content into their operations, or their positive psychological disposition toward this technology (V. Gupta, 2024). This construct has been examined within the broader technology acceptance literature, particularly in relation to models such as the Unified Theory of Acceptance and Use of Technology (UTAUT), which emphasize the role of perceived usefulness, ease of use, and social and contextual factors in shaping adoption behavior (Davis, 1989; Venkatesh et al., 2003; R. Gupta et al., 2024).

The rapid diffusion of generative AI technologies has led to the emergence of emotional AI systems that detect, interpret, and respond to human emotions. Within organizational settings, emotional AI redefines emotional labor by algorithmically monitoring and regulating employees’ emotional expressions to meet service standards (Roemmich et al., 2023). While such tools can enhance performance and customer satisfaction, they may also heighten emotional strain by limiting authenticity and autonomy. In high-demand emotional labor environments such as call centers, integrating generative or emotional AI may amplify stress, particularly when technical malfunctions occur or when algorithmic feedback conflicts with genuine emotional states (Oder & Béland, 2025).

Generative AI acceptance reflects both cognitive evaluations and emotional responses to work environments where AI technologies are increasingly present. Consistent with UTAUT, acceptance beliefs such as performance expectancy and effort expectancy may develop in advance of or alongside direct workplace use, as employees evaluate technologies based on anticipated relevance, organizational exposure, and perceived fit with their work context. This suggests that generative AI acceptance functions as a context-sensitive mechanism influencing employee responses to AI across varying levels of workplace AI exposure.

### 1.6. Emotional Intelligence

Salovey and Mayer (1990) originally defined emotional intelligence as a subset of social intelligence, referring to the ability to perceive, monitor, and regulate one’s own and others’ emotions and to use this information to guide thinking and behavior. Subsequent research distinguishes between ability-based emotional intelligence and perceived emotional intelligence, which refers to individuals’ self-assessed capacity to recognize, understand, and regulate emotions in social and organizational contexts (Wong & Law, 2002). This study adopts the perceived emotional intelligence perspective, consistent with the use of self-report measures in organizational research. Recent studies have similarly employed WLEIS as a moderator in moderated mediation designs examining behavioral outcomes in service and healthcare contexts (Yu et al., 2025).

In organizational settings, emotional intelligence has been associated with a variety of positive outcomes. Prior research shows that employees with higher emotional intelligence are better able to cope with psychological contract violations, maintain perceptions of fairness, and sustain emotional well-being (Coyle-Shapiro et al., 2019; Balogun et al., 2018). Emotional intelligence also plays an important role in emotional labor processes. Employees with higher emotional intelligence tend to manage emotional dissonance more effectively and are more likely to engage in deep acting rather than surface acting when responding to organizational display rules (Bastian et al., 2005). In this sense, emotional intelligence functions as a psychological resource that reduces burnout, strengthens resilience, and supports authentic emotional regulation in service roles (Konuk et al., 2023).

Perceived emotional intelligence may therefore function as a boundary condition that shapes the extent to which AI-related evaluations translate into surface or deep acting, a role examined further in the theoretical framework and hypothesis development sections below.

### 1.7. Theoretical Framework

The proposed model conceptualizes employees’ responses to artificial intelligence (AI) as a structured psychological process linking psychological contract orientations to emotional labor outcomes. Conservation of Resources (COR) theory (Hobfoll, 1989) explains how employees evaluate AI-related changes in terms of potential resource gains and losses, while the Theory of Planned Behavior (TPB) (Ajzen, 1991) clarifies how these evaluations translate into behavioral intentions, represented in the present model by generative AI acceptance.

Recent organizational research has similarly drawn on COR and TPB in combination, arguing that while COR theory identifies the motivational significance of resource protection, TPB provides a framework for understanding how resource-related perceptions translate into behavioral intentions and subsequent actions (Schmitt & Prasastyoga, 2024; Kim & Kim, 2024). Employees interpret AI-related changes through their psychological contract orientation, which shapes how workplace conditions and emerging technologies are evaluated. In line with COR theory, individuals assess environmental changes based on their implications for valued resources such as job security, competence, autonomy, and social support (Hobfoll, 1989). In the context of AI, this evaluation process is reflected in whether employees perceive AI as enhancing efficiency and support or as increasing uncertainty, performance pressure, and concerns about skill obsolescence (Lin & He, 2025). These evaluations do not lead to uniform outcomes, as responses vary depending on individual interpretations and available resources (Hobfoll, 1989).

These resource-based evaluations are reflected in employees’ attitudes toward AI and their levels of AI-related anxiety. The sequential ordering of these two constructs is grounded in Cognitive Appraisal Theory (Lazarus & Folkman, 1984), which holds that individuals first evaluate whether environmental conditions are threatening or beneficial, and only subsequently experience an affective response proportional to that appraisal. Within this framework, attitudes toward AI represent the cognitive appraisal stage: relatively stable evaluations of whether AI constitutes a resource gain or threat. AI anxiety represents the affective response that follows when that appraisal signals resource threat. This appraisal-to-affect direction is theoretically consistent with COR theory’s resource evaluation logic (Hobfoll, 1989) and has received empirical support in prior AI research (R. Li et al., 2025; Wang & Wang, 2022), although the strength and direction of this association may vary across different psychological contract orientations. Psychological contract orientations represent stable relational schemas that are conceptually prior to context-sensitive AI-related evaluations (Rousseau, 1995). Generative AI acceptance reflects a behavioral intention shaped by prior evaluative states, particularly attitudes toward AI, and may subsequently influence employees’ emotional labor strategies (Venkatesh et al., 2003; Grandey, 2000).

TPB provides a general framework for understanding how evaluative states translate into behavioral intentions (Ajzen, 1991). In this model, attitudes toward AI and AI-related anxiety directly link psychological contract orientations to emotional labor outcomes through serial mediation. Separately, attitudes toward AI also shape generative AI acceptance as a behavioral intention construct, which constitutes an additional pathway to emotional labor outcomes. Emotional labor is conceptualized as an intentional self-regulatory process shaped by how employees interpret their work context and role demands (Grandey, 2000; Hochschild, 1983).

The model further recognizes that these processes may not operate uniformly across employees or contract orientations. Relational orientations are more likely to involve internalized and affect-driven mechanisms, reflecting the broader socioemotional investment that characterizes such contracts. Transactional orientations, by contrast, involve more bounded exchange expectations and comparatively limited organizational attachment, which may constrain the conditions under which AI-related evaluations activate extended affective appraisal sequences. This aligns with COR theory’s emphasis on the context-dependent nature of resource appraisals (Hobfoll, 1989).

UTAUT (Venkatesh et al., 2003) grounds generative AI acceptance as a behavioral intention construct by explaining adoption through performance expectancy, effort expectancy, social influence, and facilitating conditions, which are the dimensions directly operationalized in the measurement model of the present study.

Finally, perceived emotional intelligence is conceptualized as a personal psychological resource within the COR framework that influences how employees regulate emotional responses and manage evaluative demands (Hobfoll, 1989; Wong & Law, 2002). In this model, emotional intelligence operates as a moderator that determines the strength of the indirect pathways between AI attitudes and emotional labor outcomes through generative AI acceptance.

Taken together, these frameworks do not offer parallel explanations of separate phenomena but collectively describe a single psychological adaptation process through which psychological contract orientations become associated with emotional labor strategies. The process begins with resource appraisal: employees evaluate AI-related workplace change in terms of its implications for valued resources, an appraisal shaped by the expectations embedded in their psychological contract orientation (Hobfoll, 1989). This appraisal produces a cognitive evaluation reflected in general attitudes toward AI, which, when a resource threat is perceived, is associated with a corresponding affective response in the form of AI anxiety (Lazarus & Folkman, 1984). These evaluative states shape a behavioral disposition: employees with more favorable evaluations of AI are more likely to develop an intention to engage with generative AI at work, structured through expectations of performance benefit, ease of use, social encouragement, and organizational support (Ajzen, 1991; Venkatesh et al., 2003). This acceptance-related intention is subsequently associated with whether employees regulate their emotional displays through surface acting or deep acting, a relationship moderated by perceived emotional intelligence as a personal resource that shapes the translation of behavioral intention into emotional labor strategy (Hobfoll, 1989; Wong & Law, 2002).

### 1.8. Hypothesis Development and Proposed Research Model

#### 1.8.1. Psychological Contracts and Emotional Labor

Employees who hold relational psychological contracts view their relationship with the organization as grounded in trust and long-term socioemotional exchange. Empirical evidence from long-term care facilities suggests that fulfilling relational psychological contracts strengthens employees’ emotional commitment and fosters authentic engagement in their roles (C. J. Chen et al., 2024). The reciprocity norm inherent in relational psychological contracts motivates employees to invest genuine emotional effort, consistent with deep acting as described above. Recent findings support this mechanism, showing that relational obligations promote emotional authenticity and improve the quality of emotional labor (Han et al., 2025). In service contexts, where emotional expression is a core part of performance, this sustained socioemotional commitment naturally leads to deep acting rather than surface-level emotional displays.

The hypothesis examined in this study concerns employees operating in organizational contexts where AI technologies are present to varying degrees. The proposed mechanisms linking psychological contract orientations to emotional labor outcomes through AI-related evaluations do not presuppose active or frequent AI use. Instead, these mechanisms are expected to operate among employees who perceive AI as a relevant feature of their organizational environment, regardless of the extent to which they have personally integrated these tools into their daily work practices. By contrast, transactional psychological contracts emphasize short-term, specific, and economically defined exchanges involving minimal emotion (Rousseau, 1995). As these contracts do not require a significant level of emotional investment, employees are less motivated to align their genuine emotions with organizational expectations. Instead, they rely on surface acting rather than genuine emotional alignment. This pattern is associated with emotional exhaustion and reduced service quality (Glomb & Tews, 2004). When employees perceive a breach of the transactional contract, such as a lack of supervisory support or inconsistent feedback, they may withdraw emotionally. This results in service behaviors that are mechanical and insincere, and consistent with surface acting (Han et al., 2025; Allen & Augustin, 2025).

Taken together, these patterns indicate the existence of two distinct emotional regulation pathways. Relational contracts facilitate authentic emotional alignment, while transactional contracts encourage externally regulated, imitative emotional displays.

Hence, we expect the following:

**H1.**  *Transactional psychological contract positively predicts surface acting.*

**H2.**  *Relational psychological contract predicts deep acting.*

#### 1.8.2. General Attitudes Toward AI and AI Anxiety as Serial Mediators

Artificial intelligence (AI) is increasingly embedded in work environments shaped by algorithmic control and standardized processes, often described as Digital Taylorism (Konuk et al., 2023). These conditions influence how employees evaluate AI and its implications for their work.

When transactional obligations are perceived to be violated, especially during AI-driven organizational change, employees may feel that they receive insufficient support or reciprocity from the organization. This can strengthen negative attitudes toward AI, as technological change may be seen as increasing performance pressure and job insecurity (C. J. Chen et al., 2024).

Attitudes reflect individuals’ cognitive evaluations of technological change and shape how AI-related risks and opportunities are interpreted. In this respect, AI anxiety can be understood not only as an emotional response but also as a cognitive-evaluative belief linking attitudes to subsequent behavioral outcomes (Wang & Wang, 2022). Negative attitudes toward AI may arise when employees perceive threats such as job displacement, loss of control, or ethical concerns. These evaluations can heighten uncertainty and perceived technological risk, thereby increasing AI-related anxiety. By contrast, positive attitudes toward AI may reduce uncertainty and alleviate anxiety by framing AI as useful and supportive. Empirical evidence also supports this direction, showing that attitudes toward AI predict anxiety responses (Z. Li et al., 2025).

Negative attitudes toward AI are therefore associated with higher AI anxiety, particularly when employees fear technological displacement. Employees who anticipate losing their roles to automation tend to experience greater emotional distress and threat perceptions (Akkaya et al., 2021; S. Chen & Zhao, 2025). AI-related job insecurity may also reinforce perceptions of psychological contract breach, further increasing stress and burnout (Kim et al., 2024; Moin et al., 2024). As AI anxiety increases, employees may become emotionally withdrawn and more likely to rely on surface acting, regulating only their outward emotional expressions while experiencing internal tension (Prentice et al., 2020). As noted in the theoretical framework, however, the strength of this cognitive-affective pathway may vary depending on contract orientation and the nature of resource appraisal processes involved.

Hence, we expect the following:

**H3.**  *Negative Attitude toward AI and AI Anxiety serially mediate the relationship between transactional psychological contract and surface acting.*

In contrast, relational psychological contracts reflect a commitment to long-term mutual trust. Fulfilling a relational psychological contract fosters perceptions of organizational loyalty, psychological safety and support, enhancing positive attitudes toward AI by framing technological change as an opportunity rather than a threat (Kavak, 2024; Terzi, 2020). Employees who feel valued and secure are more likely to view AI as a tool that improves efficiency and contributes to organizational success.

These positive attitudes toward AI are expected to be associated with lower levels of AI anxiety, as employees who perceive AI as beneficial and non-threatening may be less likely to interpret technological change as a resource loss, and may therefore experience lower levels of uncertainty and job displacement concerns (Wang & Wang, 2022; Akkaya et al., 2021). Lower AI anxiety is expected to support employees’ capacity for authentic emotional engagement, facilitating deep acting consistent with the socioemotional foundation of relational psychological contracts.

Hence, we expect the following:

**H4.**  *Positive attitude toward AI and AI Anxiety serially mediate the relationship between relational psychological contract and deep acting.*

#### 1.8.3. The Mediating Role of Generative AI Acceptance

The proposed mediation pathways linking general attitudes toward AI to emotional labor outcomes through generative AI acceptance are grounded in a theoretically coherent transition from a broad evaluative orientation to a more specific behavioral intention. The Theory of Planned Behavior posits that general attitudinal orientations function as cognitive antecedents of behavioral intentions toward specific objects or technologies (Ajzen, 1991; R. Gupta et al., 2024). Accordingly, individuals’ general evaluations of AI as a technological domain may shape their willingness to accept and integrate specific AI applications into their work practices, rather than directly determining complex behavioral outcomes such as emotional labor strategies. (Manresa et al., 2025; Niu & Mvondo, 2024) Generative AI was selected as the focal acceptance construct because it currently represents one of the most pervasive, cognitively demanding, and interaction-intensive forms of AI in office-based work contexts, making it a salient context in which broader AI evaluations may translate into more concrete behavioral tendencies (Liu et al., 2025; Thomas et al., 2024). In this respect, the conceptual structure progressing from broad attitudinal orientation to specific behavioral intention and subsequently to emotional labor outcomes is theoretically derived rather than implicitly assumed (Manresa et al., 2025).

Adverse attitudes toward AI, including perceptions that AI is dangerous, uncontrollable, or used unethically, can undermine employees’ confidence in AI technologies and reduce their willingness to incorporate these systems into their work practices (Schepman & Rodway, 2020; Kavak, 2024). When employees hold negative evaluations of AI, their acceptance of generative AI tools tends to decrease, limiting the extent to which these technologies are used to automate routine tasks or alleviate workload pressures. As a result, employees may continue to experience high task demands and emotional strain while still being expected to display positive and professional emotions in customer interactions.

Under such conditions, the sustained emotional demands of service roles are more likely to be managed through surface-level regulation rather than genuine emotional alignment. In this process, generative AI acceptance functions as a key mechanism linking negative attitudes toward AI to emotional labor outcomes. Lower acceptance of generative AI restricts the potential efficiency and workload-reducing benefits of AI systems, thereby maintaining emotional strain and increasing the likelihood of surface acting.

Hence, we expect the following:

**H5.**  *Generative AI acceptance mediates the relationship between negative attitude toward AI and surface acting.*

Conversely, individuals who perceive AI as beneficial and believe that it can enhance their professional functioning tend to demonstrate higher levels of generative AI acceptance. Empirical evidence from healthcare and education contexts indicates that employees who recognize the advantages of generative AI tools, such as improved efficiency, decision support, and workload reduction, also develop more favorable attitudes toward AI technologies (Tuncer & Tuncer, 2024; Cengiz & Peker, 2025). When generative AI tools are accepted and integrated into work processes, employees may delegate routine and cognitively demanding tasks to technological systems. This shift can allow employees to allocate greater cognitive and emotional resources to relational and interactive aspects of their roles.

The availability of these resources may support more authentic forms of emotional regulation. Employees who experience reduced cognitive strain and improved task efficiency may find it easier to regulate their internal emotional states and align their genuine feelings with organizational display rules. In this sense, generative AI acceptance may facilitate deep acting by enabling employees to engage more with emotional demands rather than relying on surface acting.

Hence, we expect the following:

**H6.**  *Generative AI acceptance mediates the relationship between positive attitude toward AI and deep acting.*

#### 1.8.4. Moderated Mediation Effect of Emotional Intelligence

Emotional intelligence has been identified as an important capability that facilitates individuals’ adaptation to technologically complex work environments, including those shaped by the increasing integration of AI in organizational settings. Core emotional intelligence competencies such as emotional self-control, stress tolerance, and adaptability enable employees to regulate affective responses and maintain emotional stability under demanding conditions (Akkaya et al., 2021). Individuals with higher emotional intelligence are better able to interpret emotional cues, regulate internal emotional states, and preserve psychological resources during periods of organizational transformation. Prior research also indicates that emotional intelligence training can mitigate the negative consequences of emotional labor and reduce burnout among frontline employees (Prentice et al., 2020). In addition, emotional intelligence has been shown to strengthen employees’ ability to maintain emotional balance and effectively utilize organizational resources during technological transitions (Roemmich et al., 2023).

As established in H5, the pathway from negative AI attitudes to surface acting operates through reduced generative AI acceptance, which sustains emotional strain in service interactions.

Emotional intelligence may shape how strongly this process unfolds. Employees with higher emotional intelligence possess stronger emotional regulation capabilities and are more able to translate their evaluations of workplace technologies into emotional labor strategies. Consequently, emotional intelligence may strengthen the relationship between generative AI acceptance and surface acting. When acceptance of generative AI is low, employees with higher emotional intelligence may be more likely to regulate their emotional expressions strategically in order to comply with organizational display rules. Under such conditions, employees are required to manage the discrepancy between their internal evaluations and the emotional display requirements of their roles. In this way, emotional intelligence functions as a conditional factor that shapes the indirect relationship between negative attitudes toward AI and surface acting through generative AI acceptance. Prior research supports this direction, showing that emotional intelligence does not uniformly suppress surface acting; rather, under conditions of persistent evaluative misalignment, emotionally intelligent employees may be more likely to engage in surface-level regulation as a form of deliberate compliance with organizational display norms (Karim & Weisz, 2011; Sun et al., 2025).

Hence, we expect the following:

**H7.**  *The indirect effect of a negative attitude toward AI on surface acting via generative AI acceptance becomes stronger for employees who possess higher emotional intelligence.*

Conversely, emotional intelligence may also strengthen the positive mechanisms associated with favorable attitudes toward AI. Employees who hold positive evaluations of AI technologies are generally more willing to adopt and integrate generative AI tools into their work practices. The use of such technologies can improve task efficiency and reduce routine workload, allowing employees to allocate greater cognitive and emotional resources to relational aspects of their roles.

Emotional intelligence may influence how effectively these cognitive evaluations are translated into emotional regulation strategies. Individuals with higher emotional intelligence possess stronger abilities in emotional awareness, perspective taking, and emotional regulation, which facilitate authentic emotional engagement in interpersonal interactions (Akkaya et al., 2021). When employees both accept generative AI technologies and possess high emotional intelligence, they may be better able to transform the efficiency gains associated with AI use into genuine emotional alignment with organizational display rules. In this way, emotional intelligence may strengthen the relationship between generative AI acceptance and deep acting by enabling employees to engage more authentically with the emotional demands of their work.

Hence, we expect the following:

**H8.**  *The indirect effect of positive attitude toward AI on deep acting via generative AI acceptance becomes stronger for employees with higher emotional intelligence.*

**Figure 1:**
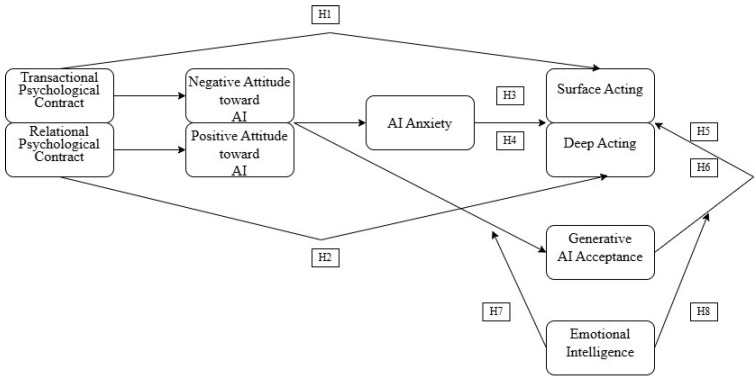
The conceptual model.

The conceptual model is presented in Figure 1, illustrating the hypothesized direct, mediating, and moderated mediating effects.

## 2. Materials and Methods

### 2.1. Measurement

All study variables were measured using established and validated scales. Generative AI acceptance was assessed with a 20-item scale developed by Yilmaz et al. (2024). Consistent with UTAUT’s conceptualization of acceptance as behavioral intention, generative AI acceptance in this study reflects employees’ evaluative disposition toward adopting generative AI tools rather than their actual usage behavior. This operationalization is particularly appropriate given that a portion of the sample reported no workplace AI use. Emotional labor was measured through the scale developed by Diefendorff et al. (2005), which was originally based on items from Grandey (2003) and Kruml and Geddes (2000), and adapted into Turkish by Basım and Beğenirbaş (2012). General attitudes toward AI were measured with the 20-item instrument created by Schepman and Rodway (2020) and later adapted by Kaya et al. (2024). The psychological contract was assessed with a 17-item scale developed by Millward and Hopkins (1998) and adapted into Turkish by Mimaroğlu (2008). AI anxiety was captured with the 16-item measure developed by Wang and Wang (2022) and adapted by Akkaya et al. (2021). Finally, emotional intelligence was measured using the Wong and Law Emotional Intelligence Scale (WLEIS), consisting of 16 items (developed by Wong & Law, 2002; adapted into Turkish by Atilla, 2012). All items were rated on a five-point Likert scale ranging from 1 (strongly disagree) to 5 (strongly agree).

### 2.2. Data Collection

The data were collected through an online survey administered via SurveyMonkey. Two filter questions were included in the survey. The first screened for working conditions: only respondents who indicated office-based work were permitted to proceed, while those selecting hybrid or remote arrangements were automatically excluded. The second differentiated respondents by AI usage: participants who reported no AI use were directed to the main questionnaire, while those who indicated AI use were asked follow-up questions about the specific tools they use and the frequency of AI use in their work context, enabling the construction of dummy variables for the analysis. The inclusion of respondents with varying AI usage profiles is theoretically grounded in the perceptual operationalization of the key constructs. Generative AI acceptance and general attitudes toward AI are conceptualized as expectation-based evaluations rather than behavioral measures of current use; employees without active workplace AI use can therefore form meaningful evaluative perceptions based on organizational exposure and general awareness of AI technologies.

Prior to participation, respondents were provided with a written informed consent statement. This statement explained that participation was voluntary, that respondents could withdraw at any time without penalty, and that the data would be used solely for academic purposes. Written informed consent was obtained electronically, with participants indicating their consent by clicking the “continue” option and proceeding to the questionnaire. Participants were reached directly through workplace contacts and by sharing the survey link on social media platforms.

The survey was distributed between 13 December 2024 and 6 May 2025. In total, 962 questionnaires were collected. During the data screening process, 93 participants were excluded because they failed the control question designed to ensure data quality. After this screening, 869 valid responses were retained for the final analysis.

The final sample consisted predominantly of female respondents (69.6%), which reflects the gender distribution in sectors such as education (30.5%), health (21.2%), and services (12.4%), where women represent the majority of employees (TÜİK, 2025). Most participants were married (56.6%), between the ages of 34 and 41 (39.6%), and held a bachelor’s degree (54.4%). In terms of tenure, 45.3% of respondents reported between 0 and 5 years of work experience.

Additionally, 79.2% of respondents reported that they use artificial intelligence technologies in general, indicating that AI tools are widely adopted in employees’ everyday lives beyond the workplace. Among these users, ChatGPT was the most frequently used tool, with 78.1% reporting its use. However, when focusing specifically on work-related use, a more differentiated pattern emerges. While a substantial proportion of respondents actively integrate AI tools into their work tasks, 13.2% indicated that they do not use AI in their professional activities. In terms of usage frequency at work, 24.4% of respondents reported using generative AI tools several times per week, 15.0% indicated daily use, and 18.1% reported monthly use.

### 2.3. Ethics Approval

This study was conducted with the approval by Gebze Technical University Human Research Ethics Committee (Approval No.: 2024/13). All participants provided informed consent prior to participation.

## 3. Results

### 3.1. Validity and Confirmatory Factor Analyses

Prior to hypothesis testing, Confirmatory Factor Analysis (CFA) was performed using AMOS 30 software to evaluate the construct validity of the measurement model. Following the guidelines suggested by Hair et al. (2019), factor loadings below 0.40 were considered insufficient for meaningful interpretation of the latent construct. Furthermore, standardized loadings below 0.30 are generally regarded as having minimal practical significance, particularly in large samples. Accordingly, items with very low loadings were removed from the model.

Within the transactional psychological contract construct, three items showed very low factor loadings: Item 2 “I prefer to work a strictly defined set of working hours.” (0.264), Item 3 “It is important not to get too involved in your job.” (0.332), and Item 4 “I expect to be paid for any overtime I do.” (0.190). In the relational psychological contract construct, item 9 “I am heavily involved in my place of work.” (0.334) also demonstrated a low factor loading. Similarly, within the negative attitude toward artificial intelligence construct, item 7 “I think artificially intelligent systems make many errors.” (0.207) showed insufficient loading. These items were removed sequentially, and the measurement model was re-estimated after each deletion to ensure that the modifications improved the model without distorting the underlying constructs. Despite the removal of several low-loading items, the retained indicators continue to represent the core conceptual dimensions of the constructs. In the case of the transactional psychological contract, the remaining items capture the limited, exchange-based, and obligation-bound nature of the employment relationship. For the relational psychological contract, the retained indicators reflect long-term commitment, reciprocity, and future-oriented expectations within the organization. Similarly, for negative attitudes toward artificial intelligence, the remaining items represent the central themes of perceived threat, risk, and concern regarding AI technologies. Accordingly, the core theoretical meaning of the constructs remains preserved.

To validate the measurement structure, a first-order confirmatory factor analysis (CFA) was conducted, incorporating all observed variables and subdimensions. The initial model demonstrated an acceptable fit to the data (χ^2^/df = 2.241, GFI = 0.812, CFI = 0.907, TLI = 0.901, RMSEA = 0.038, SRMR = 0.0420). These fit indices were confirmed to be consistent with the acceptable thresholds recommended by Hair et al. (2019) and Kline (2023), thereby substantiating the conclusion that the measurement model had achieved adequate construct validity. In addition, all standardized loadings were found to be statistically significant (*p* < 0.001), indicating satisfactory indicator reliability.

In the second stage, a hybrid CFA model was tested. In this model, generative AI acceptance, AI anxiety, and emotional intelligence were defined as second-level constructs represented by their own sub-dimensions because they were conceptualized as holistic variables in the hypothesis testing. The remaining constructs, such as emotional labor, general attitudes toward AI, and psychological contract, were maintained at the initial level. This hybrid modeling strategy was implemented to align the measurement construct with the theoretical framework and hypotheses. The mixed model demonstrated adequate fit indices (χ^2^/df = 2.163, GFI = 0.810, CFI = 0.910, TLI = 0.907, RMSEA = 0.034, SRMR = 0.0555), thereby substantiating the adequacy of the overall measurement construct.

Reliability and convergent validity were subsequently assessed using Cronbach’s alpha, composite reliability (CR), and average variance extracted (AVE). The detailed values for these statistics are reported in Table 1. Average variance extracted (AVE) values above 0.50 are generally recommended to indicate adequate convergent validity (Hair et al., 2019). However, Fornell and Larcker (1981) argue that convergent validity may still be considered acceptable when composite reliability is sufficiently high, even if the AVE value falls below 0.50. Accordingly, composite reliability values of 0.70 or higher are generally regarded as evidence of adequate construct reliability (Hair et al., 2019). Following standard scale refinement procedures, items with low factor loadings were removed to improve psychometric properties. Further item deletion beyond this threshold was deliberately avoided to preserve the conceptual breadth of each construct, as aggressive removal risks narrowing the theoretical domain the scale is intended to capture. Even after this refinement, AVE values for Positive Attitudes toward AI (AVE = 0.45), Relational Psychological Contract (AVE = 0.46), and Emotional Intelligence (AVE = 0.48) remained marginally below the recommended threshold. This pattern reflects the inherent conceptual breadth of these constructs rather than inadequate measurement. Emotional intelligence encompasses four conceptually distinct yet interrelated facets whose heterogeneity naturally limits indicator homogeneity (Wong & Law, 2002). The relational psychological contract captures open-ended, socio-emotional obligations spanning trust, loyalty, and career expectations, which do not lend themselves to high inter-item redundancy (Rousseau, 1995). Positive attitudes toward AI constitute a broad evaluative domain encompassing utility perceptions, emotional reactions, behavioral preferences, and societal expectations toward AI technologies (Schepman & Rodway, 2023). The original GAAIS validation study deliberately retained indicators with relatively modest factor loadings to preserve construct breadth and conceptual comprehensiveness, with standardized loadings ranging from 0.46 to 0.80 across two independent validation samples; the loading range observed in the present study is consistent with this documented psychometric pattern. All standardized factor loadings in the present study exceeded 0.50 across all three constructs, and CR values remained above 0.70 in each case. As an additional robustness check, Maximum Shared Variance (MSV) and Average Shared Variance (ASV) were computed for all constructs following Hair et al. (2019) and are reported in Table 1. For all three constructs with AVE below 0.50, AVE exceeded both MSV and ASV, indicating that each construct shares more variance with its own indicators than with any other construct in the model.

The discriminant validity of the constructs was determined by employing the Heterotrait-Monotrait Ratio (HTMT) method. This approach, as outlined by Henseler et al. (2015), was chosen as it is regarded as a more reliable alternative. The results of the HTMT analysis, as presented in Table 2, indicate that all ratios were found to be below the stringent threshold of 0.85. This finding confirms that the empirical separation condition between the constructs examined in the study was successfully met. Regarding the potential impact of residual measurement error on the robustness of the moderation findings, it is important to note that in regression-based models, measurement error in predictor and moderator variables produces attenuation bias in interaction effect estimates, systematically biasing them toward zero rather than away from it (Aiken et al., 1991; Kline, 2023). This attenuation occurs because measurement error increases the variance of the predictor variables, thereby reducing the relative magnitude of the estimated interaction term (Aiken et al., 1991). Accordingly, the significant moderation effects observed in the present study would be expected to represent conservative rather than exaggerated estimates. Any remaining measurement imprecision associated with the marginally reduced AVE values would be more likely to suppress than amplify the observed moderation effects. In conclusion, the overall fit and validity statistics indicate that the mixed measurement model adequately represents the hierarchical and multidimensional nature of the constructs examined.

### 3.2. Common Method Bias

To reduce the potential risk of common method bias, several procedural remedies were implemented during the survey design and data collection process. Participation was voluntary and anonymous, which helped minimize social desirability bias and evaluation apprehension (Podsakoff et al., 2003).

The questionnaire was structured in separate construct blocks, and item order was intentionally varied within some scales (e.g., psychological contract), to reduce response pattern biases and introduce a degree of psychological separation between constructs. This design also helped reduce the likelihood that respondents could infer the hypothesized relationships among variables.

In addition, an attention-check item was included in the questionnaire, and responses from participants who failed to correctly answer this item were excluded from the analysis. This procedure helped improve data quality by eliminating careless or inattentive responses.

In order to assess the potential impact of common method bias, both Harman’s single factor test and the Common Latent Factor (CLF) approach were applied, in line with the methodology established by Podsakoff et al. (2003). In the context of Harman’s single-factor test, the entirety of the measurement items was incorporated into the unrotated exploratory factor analysis. The results obtained revealed multiple factors with eigenvalues greater than one, with the first factor accounting for 16.3% of the total variance. This is below the 50% threshold, suggesting that common method bias is unlikely to be a major issue.

Furthermore, a confirmatory factor analysis model was augmented by the incorporation of a common latent factor (CLF) to statistically control for method variance. A comparison was made between the baseline model (χ^2^/df = 2.163, GFI = 0.810, CFI = 0.910, TLI = 0.907, RMSEA = 0.034, SRMR = 0.0555) and the CLF model (χ^2^/df = 2.148, GFI = 0.813, CFI = 0.912, TLI = 0.908, RMSEA = 0.036, SRMR = 0.0557). The results indicated that model fit was largely preserved across both specifications, with all observed differences remaining negligible (ΔCFI = 0.002, ΔRMSEA = 0.002, ΔSRMR = 0.0002).

Consequently, in accordance with Harman’s test results, the CLF analysis substantiates the finding that common method variance does not constitute a significant concern within the context of this study (Podsakoff et al., 2003). The detailed comparison of model fit indices is presented in Table 3.

### 3.3. Hypothesis Testing

#### 3.3.1. Testing the Main and Mediating Effects

This study employed an analytical strategy in which measurement validity and structural hypothesis testing were conducted in separate stages. Construct validity and reliability were first established through confirmatory factor analysis (CFA) in AMOS (see Section 3.1). Following common practice in organizational behavior research, composite scores representing each validated construct were subsequently incorporated into PROCESS Macro analyses for hypothesis testing purposes (Hayes et al., 2017). For the constructs operationalized as second-order latent variables in the CFA stage, namely AI anxiety, generative AI acceptance, and emotional intelligence, composite scores were calculated by averaging the corresponding first-order subdimension scores. In total, following the removal of five items with insufficient factor loadings during the CFA stage, the measurement structure comprised 94 items loading onto 18 first-order factors, including three higher-order constructs represented through second-order factor structures.

This two-stage approach was preferred over a fully latent variable SEM specification for three primary reasons. First, simultaneously estimating this factor structure together with serial mediation, simple mediation, moderated mediation pathways, and latent interaction terms within a single covariance-based SEM framework would be expected to result in substantial parameter proliferation, raising concerns about model convergence and solution admissibility. The use of CFA-validated subscale averages as composite scores is a commonly applied strategy for managing this complexity while retaining the validated construct structure (Hayes et al., 2017). Second, regression-based bootstrapping is commonly applied in conditional process analysis and enables estimation of conditional indirect effects in complex mediation and moderated mediation models (Hayes et al., 2017). Third, the use of CFA-validated composite scores in regression-based conditional process analysis is a frequently used analytical strategy in organizational behavior research (Teng et al., 2024; Cheng et al., 2025; Z. Li et al., 2025). Hayes et al. (2017) further note that estimating latent variable interactions in conditional process models remains methodologically debated, with different estimation methods potentially producing inconsistent results under assumption violations.

The choice of this analytical strategy was also guided by the primary inferential objective of the study. The present research focuses on the estimation and interpretation of conditional indirect effects linking psychological contract orientations, AI-related evaluations, generative AI acceptance, and emotional labor outcomes. While full latent-variable SEM represents an alternative analytical framework, the present model, as described above, incorporates serial mediation pathways, moderated mediation relationships, and multiple control variables alongside the higher-order construct structure established in the CFA stage. Estimating all measurement and structural parameters simultaneously within a single latent-variable model would substantially increase model complexity and parameterization. Accordingly, the present study adopted a two-stage analytical strategy in which measurement quality was first established through CFA and the hypothesized conditional process relationships were subsequently estimated using regression-based bootstrapping. This approach is consistent with prior methodological guidance regarding the analysis of mediation and conditional process models (Hayes et al., 2017; Hayes, 2022). Furthermore, estimating latent variable interactions within a moderated mediation framework typically requires specialized procedures such as latent moderated structural equations (Klein & Moosbrugger, 2000). The implementation and interpretation of these approaches in models incorporating multiple higher-order constructs, serial mediation pathways, and several control variables substantially increases analytical complexity. Accordingly, the adopted analytical strategy reflects a balance between measurement rigor established through CFA and the direct estimation of the conditional process relationships specified by the study hypotheses.

PROCESS Macro estimates observed-score mediation and moderated mediation effects using CFA-validated composite scores. Unlike a full latent-variable SEM framework, PROCESS does not explicitly model measurement error. This is acknowledged as a methodological limitation and is addressed in the Limitations section.

To examine the relationships proposed in the research model, direct effect hypotheses were tested using Model 0, while mediation hypotheses were tested using Models 4 and 6 of PROCESS Macro Version 5.0 for SPSS 30 (Hayes et al., 2017). The analyses were conducted with 10,000 bootstrap samples, and 95% bias-corrected confidence intervals were obtained. To account for differences in AI usage, two dummy variables were included as control variables. “Non-AI User” represents individuals who do not use AI at all, while “AI User–No Workplace Use” represents those who use AI but not in their work. Active workplace users were used as the reference group. The results of the main and mediation analyses are presented in Table 4.

The findings revealed that the transactional psychological contract was significantly and positively associated with surface acting (β = 0.230, *p* < 0.001), thereby supporting H1. Among the control variables, age was negatively associated with surface acting (β = −0.082, *p* = 0.010), and non-AI users reported significantly lower levels of surface acting compared to workplace AI users (β = −0.226, *p* = 0.003). Other control variables were not significant.

The relational psychological contract was significantly associated with deep acting (β = 0.134, *p* < 0.001), supporting H2. Among the control variables, age showed a marginal negative association with deep acting (β = −0.063, *p* = 0.047), while all other control variables were not statistically significant.

The serial mediation model (Model 6) was employed to examine the indirect effects of transactional psychological contract on surface acting through negative attitudes toward AI and AI anxiety. The results showed that the total indirect effect was not statistically significant (β = 0.006, BootSE = 0.005, 95% CI [−0.002, 0.018]). The specific serial indirect path through the hypothesized sequence was also not statistically significant (β = 0.000, 95% CI [−0.006, 0.004]). The direct effect remained significant (β = 0.227, *p* < 0.001). Among the control variables, age (β = −0.089, *p* < 0.01) and non-AI user status (β = −0.249, *p* < 0.01) were significantly associated with surface acting. Therefore, H3 was not supported.

A second serial mediation analysis (Model 6) was performed to investigate whether positive attitudes toward AI and AI anxiety mediated the relationship between relational psychological contract and deep acting. The results revealed a significant total indirect effect (β = 0.030, 95% CI [0.010, 0.056]). Examining the specific indirect paths, the single-mediator path through positive attitudes toward AI (Ind1: β = 0.025, 95% CI [0.010, 0.047]) was statistically significant. The path through AI anxiety only (Ind2: β = 0.016, 95% CI [−0.001, 0.036]) did not reach statistical significance. The specific serial indirect path through the hypothesized sequence (Ind3) was statistically significant but negative in direction (β = −0.010, 95% CI [−0.018, −0.004]), providing partial support for H4. The direct effect also remained significant (β = 0.104, *p* = 0.008). The indirect effect of relational psychological contract orientation on deep acting was primarily carried by the single-mediator path through positive AI attitudes (Ind1), suggesting that the resource-enhancing cognitive pathway operates independently of anxiety reduction. The specific serial path (Ind3), while statistically significant, operated in a direction opposite to that hypothesized, indicating that the full sequential mechanism did not function as theoretically predicted. None of the control variables showed significant effects in this model.

To examine the sensitivity of the proposed mediator ordering, alternative models in which AI-related anxiety preceded general AI attitudes were estimated. The specific serial indirect path was not statistically significant in the reversed-order model (Ind3: β = 0.000, 95% CI [−0.007, 0.003]), providing evidence that the hypothesized mediator sequence is not supported when the order is reversed. (see Appendix A).

Furthermore, a mediation analysis (Model 4) was conducted to test whether generative AI acceptance mediated the relationship between negative attitudes toward AI and surface acting. The results showed that generative AI acceptance significantly mediated the relationship between negative attitudes toward AI and surface acting (β = 0.026, 95% CI [0.009, 0.049]), supporting H5. Among the control variables, age was negatively associated with surface acting (β = −0.111, *p* < 0.01), while other control variables were not significant.

Finally, another mediation analysis (Model 4) examined whether generative AI acceptance mediated the relationship between positive attitudes toward AI and deep acting. Generative AI acceptance was found to mediate the relationship between positive attitudes toward AI and deep acting (β = 0.087, 95% CI [0.001, 0.176]), supporting H6. However, the indirect effect was marginally significant and should be interpreted with caution, given the near-zero lower confidence bound. None of the control variables were statistically significant in this model.

#### 3.3.2. Testing the Moderated Mediation Effect

The moderated mediation effects of Emotional Intelligence were examined using the PROCESS Macro 5.0 for SPSS (Hayes et al., 2017). The analyses were conducted with Model 14, employing 10,000 bootstrap samples and 95% bias-corrected confidence intervals.

The index of moderated mediation was significant (β = 0.038, 95% CI [0.010, 0.078]), supporting H7. Among the control variables, age was negatively associated with surface acting (β = −0.085, *p* < 0.01), whereas gender, AI usage groups, and sector were not significant. The conditional indirect effects showed that the indirect effect was not significant at low levels of emotional intelligence (β = 0.018, 95% CI [−0.003, 0.043]) but became significant at mean (β = 0.037, 95% CI [0.019, 0.063]) and high levels (β = 0.054, 95% CI [0.029, 0.089]) of emotional intelligence. These findings indicate that the indirect effect strengthens as emotional intelligence increases.

Similarly, emotional intelligence significantly moderated the indirect effect of positive attitudes toward AI on deep acting through generative AI acceptance. The index of moderated mediation was significant (β = 0.131, 95% CI [0.015, 0.244]), supporting H8. None of the control variables showed significant effects in this model. The conditional indirect effects indicated that the indirect effect was not significant at low levels of emotional intelligence (β = 0.025, 95% CI [−0.083, 0.129]) and remained non-significant at mean levels (β = 0.091, 95% CI [−0.001, 0.182]), but became significant at high levels of emotional intelligence (β = 0.148, 95% CI [0.039, 0.254]). This pattern suggests that the mediating role of generative AI acceptance emerges primarily among individuals with higher emotional intelligence. The results of moderated mediation analyses are presented in Table 5.

**Figure 2:**
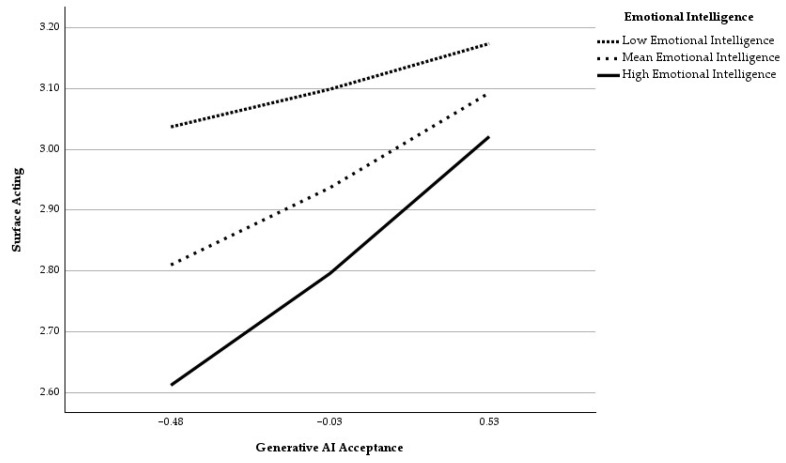
Interaction between Generative AI acceptance and emotional intelligence on surface acting.

**Figure 3:**
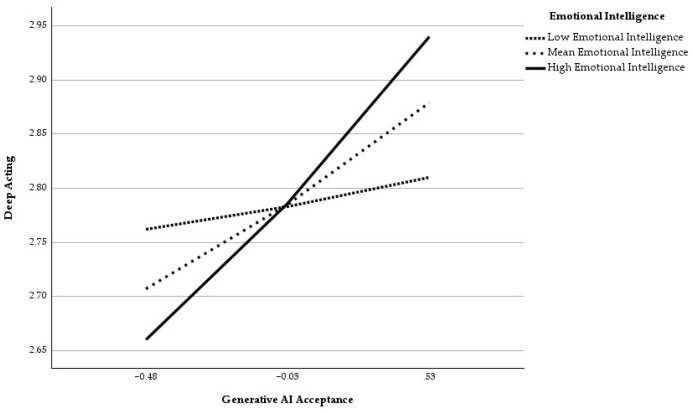
Interaction between Generative AI acceptance and emotional intelligence on deep acting.

The interaction effects are illustrated in Figure 2 and Figure 3.

To examine potential heterogeneity across AI usage groups, one-way ANOVA analyses were conducted comparing non-users (*n* = 181), users without work-related use (*n* = 91), and active workplace users (*n* = 597). Significant group differences were found for AI anxiety (F(2, 866) = 13.43, *p* < 0.001, η^2^ = 0.03). Post hoc comparisons indicated that non-users reported higher anxiety than both users without work-related use (*p* = 0.009) and active workplace users (*p* < 0.001). Transactional psychological contract scores also differed significantly across groups (F(2, 866) = 6.97, *p* < 0.001, η^2^ = 0.02), with users without work-related use scoring higher than both non-users (*p* = 0.009) and active workplace users (*p* < 0.001). For relational psychological contract (F(2, 866) = 6.43, *p* = 0.002, η^2^ = 0.02), active workplace users scored higher than both non-users (*p* = 0.046) and users without work-related use (*p* = 0.007). Generative AI acceptance showed the largest group difference (F(2, 866) = 80.67, *p* < 0.001, η^2^ = 0.16), with all three groups differing significantly from one another (all *p* < 0.001). Similarly, positive AI attitudes (F(2, 866) = 50.64, *p* < 0.001, η^2^ = 0.10) differed significantly across all group pairs (all *p* ≤ 0.001). Negative AI attitudes (F(2, 866) = 8.77, *p* < 0.001, η^2^ = 0.02) and surface acting (F(2, 866) = 5.23, *p* = 0.006, η^2^ = 0.01) differed significantly between active workplace users and non-users (*p* < 0.001 and *p* = 0.004, respectively), while no other pairwise differences were observed. No statistically significant differences were found for emotional intelligence (F(2, 866) = 0.50, *p* = 0.607, η^2^ = 0.001) or deep acting (F(2, 866) = 0.13, *p* = 0.875, η^2^ = 0.000). The results are summarized in Table 6.

Given the substantial group difference observed for Generative AI Acceptance (η^2^ = 0.157), additional multi-group CFA analyses were conducted to assess measurement invariance across the three AI usage groups following the procedures recommended by Putnick and Bornstein (2016). The results supported configural invariance (χ^2^/df = 2.106, CFI = 0.955, RMSEA = 0.036) and metric invariance (χ^2^/df = 2.105, CFI = 0.952, RMSEA = 0.036; ΔCFI = 0.003, ΔRMSEA = 0.000), consistent with the recommended threshold criteria proposed by F. F. Chen (2007). Tests of scalar invariance indicated comparatively weaker fit (χ^2^/df = 2.536, CFI = 0.929, RMSEA = 0.042; ΔCFI = 0.023, ΔRMSEA = 0.006), suggesting partial rather than full scalar invariance across groups.

## 4. Discussion

### 4.1. Major Findings and Implications

The findings of this study unveil a multifaceted psychological framework through which employees navigate work environments where AI technologies are increasingly present. Firstly, the results obtained from the study confirmed Hypotheses 1 and 2, thereby demonstrating that transactional psychological contracts are associated with surface acting, whereas relational psychological contracts are positively associated with deep acting. These findings serve to reinforce the long-standing assertion that transactional psychological contracts are associated with emotional dissonance, consistent with the characterization of such contracts as low-trust, low-support arrangements, while relational psychological contracts appear to be linked to greater psychological safety and authenticity, supporting employees’ capacity for more sincere emotional regulation (C. J. Chen et al., 2024; Grandey, 2003). This provides empirical confirmation that emotional labor strategies remain fundamentally shaped by the socio-relational quality of employment arrangements, even in technologically mediated settings. While prior research has largely explained the link between relational psychological contracts and deep acting through interpersonal trust and socioemotional exchange, the present findings suggest that this relationship also involves employees’ cognitive and emotional evaluations of emerging technologies. In organizational contexts where AI technologies are present, relational contracts appear to be associated with more favorable interpretations of AI and lower AI-related concerns, a pattern consistent with greater capacity for authentic emotional engagement in service interactions. This indicates that relational psychological contracts may be linked to emotional labor not only through traditional socio-relational mechanisms but also through technology-related cognitive–affective processes associated with how employees respond to AI-driven organizational change. It is further noted that these relational dynamics may be partly shaped by the cultural context in which the data were collected; potential cultural influences on the reported associations are discussed in the limitations section

Secondly, the results of the serial mediation process reveal a marked divergence between the two contract types. The hypothesis that H3 would be supported was not confirmed, as the cognitive-affective pathway involving negative attitudes and AI anxiety did not significantly transmit the effect of transactional psychological contracts on surface acting. One possible interpretation concerns the distinct relational characteristics of transactional psychological contracts. Transactional orientations are generally associated with comparatively limited socioemotional investment, shorter-term reciprocity expectations, and more economically bounded exchange relationships (Rousseau, 1995). Within such contexts, employees may be more likely to evaluate AI-related organizational changes in terms of immediate task-related or exchange-related implications rather than broader socio-relational or identity-based considerations. Consistent with appraisal-based perspectives on anxiety (Lazarus & Folkman, 1984), anxiety responses are more likely to emerge when organizational changes are interpreted as relevant to valued goals, identity, or longer-term well-being. In this regard, the comparatively bounded and short-term nature of transactional exchange relationships may be associated with lower engagement in extended affective appraisal processes related to AI-driven organizational change. Under such conditions, negative attitudes toward AI may not consistently translate into heightened AI anxiety in a sufficiently sequential manner to support the hypothesized serial mediation effect. Although the present study proposed this sequence based on prior research linking transactional contract conditions to heightened stress and distrust-related outcomes (J. L. Johnson & O’Leary-Kelly, 2003; Balogun et al., 2018), the current findings may indicate that the proposed pathway is more context-sensitive than initially assumed. This interpretation is also broadly consistent with emerging evidence suggesting that affective pathways linking AI-related evaluations to employee outcomes may not operate uniformly across contexts (Lin & He, 2025). Importantly, this interpretation should be regarded as exploratory rather than conclusive. The present findings do not directly establish an alternative mechanism, nor do they demonstrate the absence of AI-related evaluations among transactional employees. Rather, the non-significant serial indirect effect suggests that the proposed cognitive-affective sequence may not fully capture the ways in which transactional orientations relate to surface acting in this context.

In contrast, the results provide partial support for H4, indicating that relational psychological contract orientation is associated with deep acting primarily through positive AI attitudes. While the cognitive–affective pathway did not transmit the effects of transactional contracts, positive AI attitudes emerged as a meaningful pathway through which relational contract orientation was associated with deep acting. This asymmetry suggests that the cognitive and emotional responses to AI examined in this study may be more readily activated within relational employment contexts, although the partial nature of the serial mediation indicates that these processes may not operate uniformly across all proposed pathways. The negative direction of the specific serial indirect path (Ind3) warrants cautious interpretation within a COR framework. Deep acting is among the most resource-intensive emotional labor strategies because it requires employees to actively regulate and internalize emotional expressions rather than merely manage outward displays (Grandey, 2000). COR theory suggests that resource depletion constrains individuals’ capacity to engage in behaviors that require sustained psychological investment (Hobfoll, 1989). Consistent with this perspective, prior research has shown that emotionally demanding workplace conditions may reduce employees’ capacity to maintain effortful discretionary behaviors (Dongmo & Tanova, 2025). The present findings suggest that when AI anxiety enters the sequential pathway, its association with deep acting runs counter to the direction implied by the overall indirect effect, a pattern that may reflect resource constraints that were not directly measured in this study. Accordingly, the findings imply that the more directly supported pathway linking relational psychological contract orientation to deep acting operates through positive AI attitudes rather than through the anxiety-mediated serial route. However, because the present study did not directly measure resource depletion processes and employed a cross-sectional design, these interpretations should be regarded as exploratory rather than conclusive. Future longitudinal or experience-sampling studies may help clarify the temporal ordering and resource dynamics underlying these relationships.

Third, the analyses involving generative AI acceptance provide further insight into the mechanisms linking AI-related attitudes to emotional labor strategies. The findings support H5, indicating that generative AI acceptance mediates the relationship between negative attitudes toward AI and surface acting. Employees who hold stronger negative attitudes toward AI appear less willing to adopt generative AI tools in their work processes. This reduced acceptance may be associated with fewer efficiency gains and workload reductions that AI-supported work can provide, which in turn appears to be linked to a higher likelihood of surface acting to comply with emotional display rules (Kavak, 2024; Kim & Lee, 2025).

Furthermore, H6 was supported, indicating that generative AI acceptance mediates the relationship between positive attitudes toward AI and deep acting. Notably, the direct effect of positive attitudes toward AI on deep acting became non-significant when generative AI acceptance was included in the model, suggesting that generative AI acceptance may account for most of the association between positive AI attitudes and deep acting. However, the indirect effect was modest and longitudinal research would be needed to establish the degree of mediation more definitively. Importantly, this indirect effect emerged when control variables were incorporated into the model, particularly AI usage group membership. This finding suggests that the behavioral implications of positive attitudes toward AI may depend partly on employees’ actual exposure to AI technologies in their daily work. Employees who use AI more regularly in their work may be better positioned to act on positive evaluations of AI, such that generative AI tools are associated with lower routine workload and more authentic emotional engagement with customers and stakeholders.

Finally, the moderated mediation analyses supported H7 and H8, demonstrating that emotional intelligence strengthens this indirect pathway primarily among high-EI employees. Prior research suggests that common method variance tends to weaken rather than inflate interaction effects. Therefore, finding a significant interaction provides additional support that the effect reflects a real relationship (Siemsen et al., 2010). Employees with higher emotional intelligence appear better able to transform their cognitive evaluations of AI technologies into effective emotional regulation strategies. Emotional intelligence has been associated with more effective emotional regulation, greater preservation of psychological resources, and stronger alignment between cognitive appraisals and behavioral responses (Wong & Law, 2002; Grandey, 2000). The present finding is consistent with evidence suggesting that the relationship between emotional intelligence and surface acting is not uniformly negative. Karim and Weisz (2011) demonstrated that emotional intelligence dimensions interact with negative affectivity to amplify surface acting: employees high in self-emotional appraisal and use of emotion showed greater surface acting under conditions of high negative affect, indicating that emotional intelligence can strengthen the translation of negative evaluative states into surface-level emotional regulation. Similarly, Sun et al. (2025) found that emotional intelligence positively predicted surface acting among kindergarten teachers, attributing this to heightened sensitivity to organizational display rules among emotionally intelligent individuals. Consistent with these findings, the present results suggest that emotional intelligence functions as a translation capacity rather than a uniform suppressor of surface acting, amplifying the behavioral expression of negative technology appraisals when generative AI acceptance is constrained. In this sense, emotional intelligence emerges as a critical capability that shapes how employees emotionally adapt to work environments shaped by AI technologies.

The one-way ANOVA results provide additional context for interpreting these findings. Active workplace AI users reported significantly higher generative AI acceptance and more positive attitudes toward AI than both non-users and users without work-related use, while non-users reported higher AI anxiety than both other groups. These patterns are consistent with the broader model: employees with direct workplace exposure to AI appear to develop more favorable evaluative orientations, which in turn are associated with more adaptive emotional labor strategies. Notably, no significant group differences were found for deep acting or emotional intelligence, suggesting that these individual-level processes operate relatively independently of AI usage status. Additional measurement invariance analyses further informed the interpretation of group differences in Generative AI Acceptance. Although metric invariance supported the comparability of the construct across AI usage groups, the comparatively weaker scalar invariance findings suggest that employees with differing levels of AI exposure may not respond to all AI acceptance items in fully equivalent ways. Accordingly, observed mean-level differences may partly reflect differences in response framing across groups rather than attitudinal differences alone. This pattern is consistent with the broader measurement invariance literature (Putnick & Bornstein, 2016).

The findings indicate that emotional labor in work contexts characterized by growing AI presence is shaped by the combined influence of psychological contracts, technology-related evaluations, and individual emotional competencies. Together, these elements form a complex psychological structure through which employees interpret and respond to technological transformation in contemporary service environments.

### 4.2. Theoretical Implications

The findings of this study offer several theoretical contributions by integrating psychological contract theory, emotional labor research, and technology-related attitudes within a unified framework suitable for contemporary work environments where AI is increasingly present. First, the results extend psychological contract theory by demonstrating that relational and transactional contracts are associated with distinct psychological processes in organizational contexts where AI technologies are present. While prior research has largely examined psychological contracts within conventional organizational structures, the present study shows that these relational dynamics remain influential in technologically transforming workplaces. More importantly, the findings suggest that the association between relational contracts and emotional labor outcomes appears to be partially explained by employees’ cognitive and emotional interpretations of AI technologies. This indicates that psychological contract theory may need to be expanded to incorporate employees’ responses to technological transformation and AI-related uncertainty.

Second, the study contributes to emotional labor theory by demonstrating that employees’ emotional regulation strategies are shaped not only by interpersonal demands and organizational display rules but also by their evaluations of technological systems. By linking attitudes toward AI, AI anxiety, and generative AI acceptance to surface and deep acting, the findings suggest that emotional labor in contemporary workplaces may also reflect technology-related antecedents. In this sense, emotional labor may be conceptualized as a hybrid process influenced simultaneously by relational, psychological, and technological forces.

Third, the study advances the emerging literature on AI-related workplace psychology by providing empirical evidence for a cognitive–affective sequence through which employees interpret technological change. Critically, this sequence was supported for relational but not transactional contract orientations, indicating that the cognitive–affective pathway from AI attitudes to anxiety to emotional labor is contract-orientation dependent. This asymmetry contributes to the integration of previously fragmented research streams by showing that the mechanism through which AI evaluations translate into behavioral outcomes is not uniform but contingent on the relational quality of the employment arrangement.

Fourth, the results contribute to technology acceptance research by highlighting the behavioral implications of generative AI acceptance within emotionally demanding work contexts. While UTAUT primarily emphasizes perceived usefulness and ease of use as drivers of adoption, the present findings suggest that acceptance processes are also embedded in broader emotional and relational dynamics. In particular, the findings indicate that negative evaluations of AI appear to be more strongly associated with behavioral outcomes than positive evaluations, thereby providing further insight into asymmetrical effects frequently discussed in technology adoption research.

Fifth, the study contributes to the literature on emotional intelligence by demonstrating its role in shaping how employees translate technology-related cognitions into emotional labor strategies. Rather than functioning solely as a protective psychological resource that buffers stress, emotional intelligence appears to strengthen the behavioral expression of both positive and negative evaluations of AI technologies. This finding suggests that emotional intelligence plays a critical role in facilitating employees’ emotional adaptation to technologically complex work environments.

Finally, the study contributes to the theoretical positioning of artificial intelligence within organizational research. Previous studies have often treated AI primarily as a contextual factor within which traditional psychological processes occur. In contrast, the present study conceptualizes AI as a theoretically embedded explanatory element that shapes employees’ cognitive, emotional, and behavioral responses. This perspective is supported by the domain-specificity of the constructs employed: AI anxiety captures concerns qualitatively distinct from general technology stress (Wang & Wang, 2022), general AI attitudes reflect broader societal and ethical evaluations rather than tool-specific perceptions, and generative AI acceptance introduces role-boundary and identity considerations absent from standard adoption models. Together, these constructs make AI a theoretically active element in the model rather than a contextual backdrop.

Taken together, these distinctions indicate that AI-related variables in the present model are not interchangeable with generic organizational stress or technology adoption constructs. Due to its autonomy, decision-making capacity, learning potential, and ability to interact with humans, artificial intelligence appears to be associated with how employees interpret social exchange relationships, psychological contract fulfillment or breach, and manage emotional labor strategies such as deep acting or surface acting. In this sense, AI functions not merely as a contextual backdrop but as a theoretically active force that structures the psychological mechanisms examined in this study.

### 4.3. Practical Implications

The findings of this study offer several practical insights for organizations integrating AI into service environments. Firstly, the divergent effects of transactional and relational psychological contracts suggest that organizations should prioritize relational employment practices. Such practices include transparent communication, long-term developmental support, and recognition systems. Such practices have been linked in prior research to deeper acting and lower emotional dissonance, and the present findings are consistent with this pattern. Furthermore, the cultivation of relational climates may support employees’ responses to technological change, given the observed associations between relational contract orientations and lower AI-related anxiety and more positive attitudes toward AI.

Secondly, the substantial mediating function of generative AI acceptance in the negative pathway signifies that training programs should not exclusively concentrate on technical competencies. Instead, they should also address concerns regarding surveillance, job displacement, and loss of autonomy. The communication of the tangible benefits of AI, including the reduction in workload and the enhancement of task efficiency, may facilitate the translation of acceptance into healthier emotional labor strategies among employees.

Thirdly, the moderating effects of emotional intelligence highlight the importance of incorporating EI-enhancing interventions into organizational development initiatives. It is submitted that workshops focusing on emotional regulation, stress tolerance, and adaptive coping may strengthen employees’ capacity to navigate AI-related uncertainty while maintaining authentic emotional engagement in customer-facing roles.

Finally, it is imperative for organizations to regard the increasing presence of AI in work environments not solely as a technological transition, but also as a psychological and emotional transformation. Human-centered implementation strategies are consistent with minimizing unintended emotional burdens and supporting both employee well-being and service quality, as suggested by the present associations between relational orientations and more adaptive emotional labor.

### 4.4. Limitations and Future Research

The present study is subject to several limitations, which nevertheless give rise to significant avenues for future inquiry. First, the cultural context of Türkiye warrants interpretive attention in light of the relational and affective mechanisms central to the proposed model. Türkiye is characterized by relatively high power distance and vertical collectivist orientations in workplace relations, which may shape how employees interpret psychological contract expectations and respond to organizationally introduced technologies (Konuk et al., 2023; Kaya et al., 2024). In higher power distance contexts, employees may be more likely to accept managerial and institutional authority, including authority embedded in AI-driven systems, with comparatively lower levels of overt resistance (Cheng et al., 2025; Konuk et al., 2023). This, in turn, may influence the threat appraisals underlying AI anxiety and affect the strength of the mediation pathways examined in the present study. Collectivist cultural norms emphasizing relational harmony and interpersonal authenticity may also shape the expression of emotional labor strategies. Deep acting may be more culturally internalized among Turkish employees as a norm of interpersonal conduct rather than solely as an organizationally imposed requirement (Konuk et al., 2023). Consistent with this perspective, empirical findings from Turkish organizational contexts suggest that employees may hold relatively pragmatic and favorable evaluations of AI as a performance-enhancing tool, while job displacement concerns may be comparatively less salient than those reported in some Western samples (Kavak, 2024). These cultural dynamics do not invalidate the proposed mechanisms; however, they do suggest that the direction and magnitude of the reported associations may partly reflect the specific cultural and institutional context in which the data were collected. Replication across culturally diverse organizational settings would therefore be valuable for assessing the broader generalizability of the findings.

Secondly, a cross-sectional research design limits the ability to infer temporal or causal relationships among psychological contracts, AI-related evaluations, and emotional labor strategies. Because all variables were measured at a single point in time, the temporal ordering of the proposed relationships cannot be definitively established. Given that the integration of AI within organizations is an evolving process, longitudinal and panel designs would facilitate the observation of how employee attitudes, anxieties, and behavioral responses unfold over time. Future research could further examine the causal relationship between attitudes toward AI and AI-related anxiety. While the present study conceptualizes attitudes as antecedents of AI anxiety, alternative causal sequences may also be possible. Additionally, the psychological contract scale employed in this study (Millward & Hopkins, 1998) was developed prior to the widespread adoption of AI in organizational contexts. Although the relational and transactional dimensions of the scale remain conceptually stable across settings, the measure may not fully capture contractual expectations that are specific to AI-driven work arrangements, which constitutes a limitation of the present measurement approach.

Thirdly, while the study encompasses a broad spectrum of psychological and emotional mechanisms, it does not fully account for contextual variables such as organizational culture, leadership style, the pace of technological change, or role-specific emotional labor demands. Emotional display requirements are not uniformly distributed across office-based occupations, and different roles may involve varying levels of interpersonal interaction and emotional regulation demands. Although the sector was statistically controlled in all analyses and the sample primarily consisted of office-based employees from education, health, and service sectors, the study did not directly assess occupation-specific emotional labor intensity or customer interaction requirements. It is therefore recommended that future research incorporate these structural and contextual factors to develop a more comprehensive understanding of how organizational ecosystems shape responses to AI. In addition, future studies may consider developing or adapting psychological contract measures that explicitly capture employees’ expectations and perceived obligations in AI-driven organizational environments. A further interpretive boundary concerns the ecological validity of the proposed mechanisms for respondents without direct workplace AI experience. Across the model, AI-related perceptual variables are used to predict actual workplace behaviors, namely surface acting and deep acting, that occur in real service interactions. General attitudes toward AI and AI anxiety are broad cognitive-evaluative and affective responses that may develop through indirect channels such as organizational discourse, media exposure, and general societal awareness of AI, without requiring sustained direct workplace use. Generative AI acceptance, however, involves more context-specific evaluations, including performance expectancy, effort expectancy, and facilitating conditions, which may rest on weaker experiential grounding for respondents without direct workplace AI use. This construct also exhibited the largest group differences in the present study (η^2^ = 0.157) and only partial scalar invariance across AI usage groups, suggesting that its measurement properties may not be fully equivalent across employees with differing levels of direct AI exposure. Accordingly, the pathways involving generative AI acceptance as a mediating variable, particularly those linking it to emotional labor outcomes, should be interpreted with caution. Direct workplace AI experience should be understood as a meaningful boundary condition of the present findings. Future research should re-examine the proposed mechanisms, particularly those involving generative AI acceptance, in samples composed exclusively of employees with substantial workplace AI experience, and establish stronger measurement equivalence across AI usage groups when formal group comparisons are intended.

Fourthly, a further interpretive consideration concerns the predominantly female composition of the sample. Prior research has reported gender differences in AI anxiety within certain occupational contexts, suggesting that these demographic characteristics warrant attention. In the present study, gender was included as a control variable in all analyses and was not statistically significant across the estimated models, with confidence intervals consistently encompassing zero. Although this pattern does not indicate a strong direct effect of gender on the focal relationships examined here, the findings may nevertheless partly reflect gender-related dynamics associated with AI evaluations and emotional labor processes. Accordingly, the results may not fully generalize to gender-balanced or male-dominant organizational contexts. Future research should therefore examine gender more explicitly as a potential boundary condition or moderator of the proposed mediation pathways.

Fifthly, the study focuses on general attitudes toward AI and AI anxiety without distinguishing between the various AI systems in use in workplaces. Employees may exhibit varied responses to decision-support AI, workflow automation, and emotional AI technologies. An examination of these distinctions may yield technology-specific pathways that influence emotional labor.

Sixthly, PROCESS-based observed-score estimation does not explicitly account for measurement error as a full latent-variable SEM would. As Kline (2023) notes, PROCESS macros assume zero measurement error in causal variables. Accordingly, regression-based indirect effect estimates may carry some degree of bias attributable to residual measurement error, which is a recognized limitation of observed-score approaches acknowledged by Hayes et al. (2017). The CFA stage established the measurement prerequisites for composite construction; however, CFA fit indices and composite reliability values do not eliminate the possibility of measurement error bias in the estimated indirect effects. Future research employing full latent-variable SEM with explicit measurement error modeling would strengthen the robustness of these findings.

Finally, emotional intelligence was conceptualized as an individual difference variable, yet emotional intelligence can be enhanced through targeted interventions. It is recommended that future experimental or field-based research investigate the following hypothesis: that emotional intelligence training programs can strengthen employees’ adaptive responses to AI-driven transformation and reduce emotional strain in service roles.

## Figures and Tables

**Table 1:** Measurement model results.

Construct (Scale)	Sub-Dimension	No. of Items	Std. Loadings Range	AVE	CR	MSV	ASV	Cronbach’s α
Generative AI Acceptance (Second-Order)		20	0.52–0.85	0.58	0.84	0.411	0.085	0.923
Generative AI Acceptance	Performance Expectancy	7	0.76–0.86	0.64	0.93	0.453	0.086	0.904
	Effort Expectancy	5	0.82–0.93	0.81	0.95	0.372	0.083	0.939
	Facilitating Conditions	3	0.66–0.86	0.61	0.82	0.372	0.066	0.693
	Social Influence	5	0.83–0.91	0.78	0.95	0.222	0.040	0.930
Emotional Labor	Surface Acting	6	0.80–0.88	0.71	0.94	0.050	0.014	0.917
	Deep Acting	4	0.84–0.91	0.78	0.94	0.044	0.011	0.904
General Attitudes toward AI	Positive Attitudes	12	0.51–0.78	0.45	0.91	0.411	0.093	0.882
	Negative Attitudes	7	0.55–0.85	0.54	0.89	0.436	0.095	0.855
Psychological Contract	Transactional Contract	5	0.53–0.80	0.54	0.85	0.097	0.012	0.787
	Relational Contract	8	0.57–0.81	0.46	0.87	0.097	0.029	0.839
AI Anxiety (Second-Order)		16	0.65–0.88	0.66	0.89	0.493	0.093	0.933
AI Anxiety	Learning Anxiety	5	0.85–0.96	0.86	0.97	0.180	0.069	0.957
	Job Replacement Anxiety	4	0.65–0.91	0.70	0.90	0.531	0.101	0.853
	Socio-technical Blindness	4	0.76–0.87	0.68	0.90	0.531	0.106	0.846
	AI Configuration Anxiety	3	0.96–0.97	0.94	0.98	0.452	0.094	0.968
Emotional Intelligence (Second-Order)		16	0.55–0.75	0.48	0.78	0.049	0.020	0.872
Emotional Intelligence	Self-Emotional Appraisal	4	0.81–0.90	0.74	0.92	0.181	0.031	0.878
	Others’ Emotional Appraisal	4	0.60–0.96	0.57	0.84	0.078	0.013	0.850
	Use of Emotion	4	0.78–0.90	0.70	0.90	0.184	0.035	0.854
	Regulation of Emotion	4	0.76–0.90	0.73	0.92	0.184	0.029	0.873

**Table 2:** HTMT result.

Variable	GAIA	SA	DA	PAAI	NAAI	TPC	RPC	AIA	EI
GAIA	.	.	.	.	.	.	.	.	.
SA	0.136	.	.	.	.	.	.	.	.
DA	0.137	0.234	.	.	.	.	.	.	.
PAAI	0.757	0.180	0.128	.	.	.	.	.	.
NAAI	0.299	0.101	0.108	0.344	.	.	.	.	.
TPC	0.055	0.258	0.065	0.075	0.064	.	.	.	.
RPC	0.234	0.102	0.124	0.186	0.080	0.454	.	.	.
AIA	0.359	0.045	0.160	0.431	0.813	0.067	0.099	.	.
EI	0.326	0.208	0.085	0.189	0.135	0.185	0.272	0.246	.

Note: GAIA: Generative AI acceptance; SA: Surface acting; DA: Deep acting; PAAI: Positive attitudes toward AI; NAAI: Negative attitudes toward AI; TPC: Transactional psychological contract; RPC: Relational psychological contract; AIA: AI anxiety; EI: Emotional intelligence.

**Table 3:** Fit indices for the measurement models.

	χ^2^	D*f*	CFI	TLI	RMSEA
Single-factor model	46,470.942	4277	0.229	0.212	0.107
Six-factor model	9145.863	4228	0.910	0.907	0.034
ULCMF	9023.407	4201	0.912	0.908	0.036

Note: ULCMF = unmeasured latent common method factor.

**Table 4:** Results of main and mediation analyses.

Path	Direct Effect (β)	Indirect Effect (β)	T	95% Confidence Interval	Supported
**H1:** Transactional Psychological Contract → Surface Acting	0.230 ***		6.309	[0.158, 0.301]	Supported
**Control Variables**					
Gender	0.020		0.288	[−0.114, 0.153]	
Age	−0.082		−2.580	[−0.145, −0.020]	
Non-AI User	−0.226		−2.931	[−0.377, −0.075]	
AI User, No Workplace Use	−0.085		−0.827	[−0.285, 0.116]	
Sector	−0.010		−1.027	[−0.029, 0.009]	
**H2:** Relational Psychological Contract → Deep Acting	0.134 **		3.373	[0.056, 0.212]	Supported
**Control Variables**					
Gender	0.026		0.388	[−0.107, 0.160]	
Age	−0.063		−1.990	[−0.126, −0.001]	
Non-AI User	0.019		0.240	[−0.134, 0.172]	
AI User, No Workplace Use	−0.010		−0.100	[−0.212, 0.191]	
Sector	−0.015		−1.549	[−0.034, 0.004]	
**H3**	0.224 ***				Not Supported
Total Indirect Effect		0.006		[−0.002, 0.018]	
Ind1: Transactional PC → Negative Attitude toward AI → Surface Acting		0.006		[−0.001, 0.021]	
Ind2: Transactional PC → AI Anxiety → Surface Acting		0.000		[−0.004, 0.006]	
**Ind3 (Specific serial path): Transactional PC → Negative Attitude toward AI → AI Anxiety → Surface Acting**		0.000		[−0.006, 0.004]	
**Control Variables**					
Gender	0.013		0.188	[−0.123, 0.149]	
Age	−0.089		−2.791	[−0.152, −0.027]	
Non-AI User	−0.249		−3.199	[−0.402, −0.096]	
AI User, No Workplace Use	−0.088		−0.862	[−0.288, 0.112]	
Sector	−0.008		−0.883	[−0.027, 0.010]	
**H4**	0.104 **				Partial Supported
Total Indirect Effect		0.030 **		[0.010, 0.056]	
Ind1: Relational PC → Positive Attitude toward AI → Deep Acting		0.025 **		[0.010, 0.047]	
Ind2: Relational PC → AI Anxiety → Deep Acting		0.016		[−0.001, 0.036]	
**Ind3 (Specific serial path): Relational PC → Positive Attitude toward AI → AI Anxiety → Deep Acting**		−0.010		[−0.018, −0.004]	
**Control Variables**					
Gender	−0.009		−0.132	[−0.143, 0.125]	
Age	−0.058		−1.846	[−0.119, 0.004]	
Non-AI User	0.061		0.766	[−0.096, 0.218]	
AI User, No Workplace Use	0.037		0.369	[−0.161, 0.236]	
Sector	−0.015		−1.549	[−0.033, 0.004]	
**H5:** Negative Attitude toward AI → Generative AI Acceptance → Surface Acting		0.026 **		[0.009, 0.049]	Supported
**Control Variables**					
Gender	−0.009		−0.124	[−0.144, 0.126]	
Age	−0.111		−3.452	[−0.175, −0.048]	
Non-AI User	−0.147		−1.748	[−0.313, 0.018]	
AI User, No Workplace Use	0.034		0.324	[−0.170, 0.238]	
Sector	−0.001		−0.115	[−0.020, 0.018]	
**H6:** Positive Attitude toward AI → Generative AI Acceptance → Deep Acting		0.087 **		[0.001, 0.176]	Supported
**Control Variables**					
Gender	0.044		0.644	[−0.090, 0.179]	
Age	−0.042		−1.325	[−0.105, 0.020]	
Non-AI User	0.118		1.403	[−0.047, 0.282]	
AI User, No Workplace Use	0.013		0.125	[−0.190, 0.215]	
Sector	−0.012		−1.224	[−0.031, 0.007]	

Note: ** *p* < 0.01, *** *p* < 0.001.

**Table 5:** Results of moderated mediation analyses.

Path			β	t	95% Confidence Interval (Boot LLCI, ULCI)		Supported
**Moderated Mediation Effect (Model 14)**							
**H7:** Emotional Intelligence × Negative Attitude toward AI → Generative AI Acceptance → Surface Acting			0.038 **		[0.010, 0.078]		Supported
**Control Variables**							
Gender			−0.012	−0.172	[−0.145, 0.121]		
Age			−0.085	−2.663	[−0.148, −0.022]		
Non-AI User			−0.097	−1.162	[−0.261, 0.067]		
AI User, No Workplace Use			0.069	0.678	[−0.132, 0.271]		
Sector			−0.002	−0.245	[−0.021, 0.016]		
**H8:** Emotional Intelligence × Positive Attitude toward AI → Generative AI Acceptance → Deep Acting			0.131 *		[0.015, 0.244]		Supported
**Control Variables**							
Gender			0.048	0.699	[−0.086, 0.182]		
Age			−0.039	−1.207	[−0.102, 0.024]		
Non-AI User			0.116	1.380	[−0.049, 0.282]		
AI User, No Workplace Use			0.022	0.215	[−0.180, 0.225]		
Sector			−0.012	−1.254	[−0.031, 0.007]		
**Conditional Indirect Effects (Moderated Mediation)**							
**Moderator Level (Emotional Intelligence)**	**H7: Surface Acting**				**H8: Deep Acting**		
	**Indirect** **Effect (β)**	**BootSE**	**95% CI** **(BootLLCI, BootULCI)**		**Indirect** **Effect (β)**	**BootSE**	**95% CI** **(BootLLCI, BootULCI)**
Low (−1 SD)	0.018	0.012	[−0.003, 0.043]		0.025	0.054	[−0.083, 0.129]
Mean	0.037	0.011	[0.019, 0.063]		0.091	0.047	[−0.001, 0.182]
High (+1 SD)	0.054	0.015	[0.029, 0.089]		0.148	0.055	[0.039, 0.254]

Note: * *p* < 0.05, ** *p* < 0.01.

**Table 6:** One-way ANOVA results: Group comparisons by AI usage status.

Variable	F(2, 866)	*p*	η^2^	Non-User vs. Work User	Non-User vs. User-No Work	Work User vs. User-No Work
Transactional PC	6.965	0.001	0.016	0.001	0.009	0.894
Relational PC	6.434	0.002	0.015	0.046	0.513	0.007
Positive AI attitudes	50.642	0.001	0.105	0.001	0.001	0.001
Negative AI attitudes	8.768	0.001	0.020	0.001	0.064	0.826
AI anxiety	13.434	0.001	0.030	0.001	0.009	0.863
Deep acting	0.134	0.875	0.000	0.939	0.985	0.897
Surface acting	5.228	0.006	0.012	0.004	0.087	1.000
GenAI acceptance	80.666	0.001	0.157	0.001	0.001	0.001
Emotional intelligence	0.500	0.607	0.001	0.585	0.799	1.000

Note. Groups: Non-user (*n* = 181), User-No Work Use (*n* = 91), Work User (*n* = 597). Pairwise comparison columns report post hoc *p* values; values in gray indicate non-significant differences (*p* > 0.05). Post-hoc comparisons used Games-Howell for GenAI acceptance and Positive AI attitudes (Levene *p* < 0.05); Tukey HSD was applied for all remaining variables.

## Data Availability

The data that support the findings of this study are available from the corresponding authors upon reasonable request.

## Abbreviations

The following abbreviations are used in this manuscript:

AI	Artificial intelligence
COR	Conservation of Resources Theory
TPB	Theory of Planned Behavior
TAM	Technology Acceptance Model
UTAUT	Unified Theory of Acceptance of Technology
ULCMF	Unmeasured latent common method factor
GAIA	Generative AI acceptance
SA	Surface acting
DA	Deep acting
PAAI	Positive attitudes toward AI
NAAI	Negative attitudes toward AI
TPC	Transactional psychological contract
RPC	Relational psychological contract
AIA	AI anxiety
EI	Emotional intelligence

## Appendix A

**Table A1:** Alternative Mediator Ordering for H3 and H4.

Path	Direct Effect (β)	Indirect Effect (β)	t	95% Confidence Interval	Supported
**Alternative H3:** Transactional PC → AI Anxiety → Negative Attitude toward AI → Surface Acting	0.224 ***	0.006		[−0.002, 0.018]	Not Supported
Ind1: Transactional PC → AI Anxiety → Surface Acting		0.000		[−0.005, 0.004]	
Ind2: Transactional PC → Negative Attitude toward AI → Surface Acting		0.005		[0.000, 0.018]	
Ind3: Transactional PC → AI Anxiety → Negative Attitude toward AI → Surface Acting		0.000		[−0.004, 0.008]	
**Control Variables**					
Gender	0.013		0.188	[−0.123, 0.149]	
Age	−0.089		−2.791	[−0.152, −0.027]	
Non-AI User	−0.249		−3.199	[−0.402, −0.096]	
AI User, No Workplace Use	−0.088		−0.862	[−0.288, 0.112]	
Sector	−0.008		−0.883	[−0.027, 0.010]	
**Alternative H4:** Relational PC → AI Anxiety → Positive Attitude toward AI → Deep Acting	0.104 **	0.030 **		[0.010, 0.056]	Not Supported
Ind1: Relational PC → AI Anxiety → Deep Acting				[−0.012, 0.025]	
Ind2: Relational PC → Positive Attitude toward AI → Deep Acting				[0.012, 0.048]	
Ind3: Relational PC → AI Anxiety → Positive Attitude toward AI → Deep Acting				[−0.007, 0.003]	
**Control Variables**					
Gender	−0.009		−0.132	[−0.143, 0.125]	
Age	−0.058		−1.846	[−0.119, 0.004]	
Non-AI User	0.061		0.766	[−0.096, 0.218]	
AI User, No Workplace Use	0.037		0.369	[−0.161, 0.236]	
Sector	−0.015		−1.549	[−0.033, 0.004]	

Note: ** *p* < 0.01, *** *p* < 0.001.
